# Gap Junctional Interaction of Endothelial Progenitor Cells (EPC) with Endothelial Cells Induces Angiogenic Network Formation In Vitro

**DOI:** 10.3390/ijms26104827

**Published:** 2025-05-18

**Authors:** Christina Buchberger, Petra Kameritsch, Hanna Mannell, Heike Beck, Ulrich Pohl, Kristin Pogoda

**Affiliations:** 1 Physiology, Institute of Theoretical Medicine, Faculty of Medicine, University of Augsburg, 86159 Augsburg, Germany; christina.steininger@med.uni-augsburg.de (C.B.); hanna.mannell@med.uni-augsburg.de (H.M.); 2 Walter Brendel Centre of Experimental Medicine, University Hospital, Ludwig-Maximilians-University, 81377 Munich, Germany; kameritsch@lmu.de; 3 Walter Brendel Centre of Experimental Medicine, Biomedical Center Munich, Ludwig-Maximilians-University, 82152 Planegg, Germany; heike.beck@med.uni-muenchen.de (H.B.); upohl@lmu.de (U.P.)

**Keywords:** gap junction, connexin, angiogenesis, endothelial cells, endothelial progenitor cells

## Abstract

Endothelial progenitor cells (EPC) are considered to support neovascularization and endothelial repair by being incorporated into newly formed or injured vessels and by improving vascularization in a paracrine manner by secreting proangiogenic factors. Here, we studied the role of gap junctional communication between EPC and endothelial cells in long-term co-cultures in vitro. The cultivation of endothelial cells together with mouse embryonic EPC (E 7.5) induced the spontaneous formation of angiogenic networks after 3–6 days consisting of both cell types, but not in the respective monocultures, whereas their respective cultivation on a basement matrix induced the formation of tube-like structures, as expected. The angiogenic network formation could not be mimicked by the incubation of endothelial cells with supernatants of EPC only. We therefore hypothesized that direct interaction and cell-cell communication is required to induce the angiogenic network formation in co-cultures with endothelial cells. Expression analysis demonstrated expression of the gap junctional protein connexin 43 (Cx43) in EPC. Moreover, dye injection studies as well as FACS analysis identified gap junctional communication between endothelial cells and EPC. The inhibition of gap junctions by pharmacological blockers significantly reduced the angiogenic network formation, confirming that gap junctional communication between both cell types is required for this process.

## 1. Introduction

Endothelial progenitor cells (EPC), the precursor cells of endothelial cells (EC), have a strong angiogenic potential and are recruited from the bone marrow into the blood stream during repair and remodeling processes of the vascular endothelium after injury and inflammation [1,2]. They contribute to neovascularization during ischemia or tumor growth by promoting angiogenesis [3,4] and are incorporated into a functional microvasculature in vivo. This incorporation can also be observed in the angiogenic tube formation of EC in vitro [4]. Interestingly, they can exert their pro-angiogenic function either directly via differentiation into mature EC and incorporation during vessel regeneration and angiogenesis, or in a paracrine manner via the release of proangiogenic growth factors and cytokines [3,5]. EPCs secrete various growth factors such as Vascular Endothelial Growth Factor (VEGF), Platelet-Derived Growth Factor (PDGF) or angiogenin [3]. These molecules stimulate existing endothelial cells and other supporting cells in the tissue to proliferate, migrate, and form new blood vessels without necessarily integrating into the vessel walls themselves. In addition to growth factors, EPCs release cytokines like Tumor Necrosis Factor-alpha (TNF-α), which modulate inflammation [6]. For the incorporation into a vascular network and the contribution to vessel repair processes and angiogenesis, they are in direct contact with the endothelium. However, how EPC and EC communicate within this context and what impact this has on the formation of new vessels has not yet been investigated. Considering their direct contact with EC, communication between EPC and EC may occur via gap junctions. However, whether a direct gap junctional communication between neighboring EC and EPC takes place during angiogenesis and if this contributes to their incorporation during vessel repair and angiogenesis has not been examined so far.

Gap junctions are clusters of transmembranous channels, which are formed by docking of two connexin hemichannels, each consisting of six subunits of connexin (Cx) proteins, of neighboring cells. Gap junctions enable a direct intercellular communication via the exchange of ions and small molecules up to 1.2 kDa [7,8]. Cx constitute a large family of proteins with 21 members in human and 20 in mouse and have a tissue specific expression [9]. In the vasculature there are four connexins expressed with Cx37, Cx40 and Cx43 in EC and Cx43 and Cx45 in vascular smooth muscle cells [10]. The most ubiquitously found Cx is Cx43 [11], which is also expressed in mouse embryonic EPC [12]. Recently, we demonstrated that Cx43 promotes endothelial cell migration and angiogenesis [13], which is in line with other studies showing that Cx43 is associated with an increased angiogenesis [14,15,16,17].

The aim of our study was to investigate if EPC form functional gap junctions with EC and the impact of Cx43 containing gap junctions for the EPC incorporation and angiogenic network formation in long-term co-cultures with mature EC in vitro. Therefore, the well-characterized murine embryonal EPC line (T17b-EPC) isolated at E7.5 of mouse development, which has been initially characterized by Hatzopoulos et al. [18] was used in this study. These cells display robust growth properties and have been shown to be positive for early endothelial markers, differentiate to EC, and be incorporated into vascular networks [4,19]. Furthermore, these cells enhance vascularization and possess an angiogenic potential in different in vitro and in vivo studies [3,4,20,21].

Here, we provide experimental evidence that co-cultivation of mature EC with embryonic EPC induces a spontaneous angiogenic network formation without an angiogenic matrix and that this is dependent on the formation of functional gap junction channels. This indicates that a direct interaction of EC with EPC and their gap junctional communication plays an important role for the EPC incorporation during vascular remodeling processes like vessel repair and angiogenesis.

## 2. Results

### 2.1. EPC Show Angiogenic Activity Only in Co-Culture with EC

**Figure 1:**
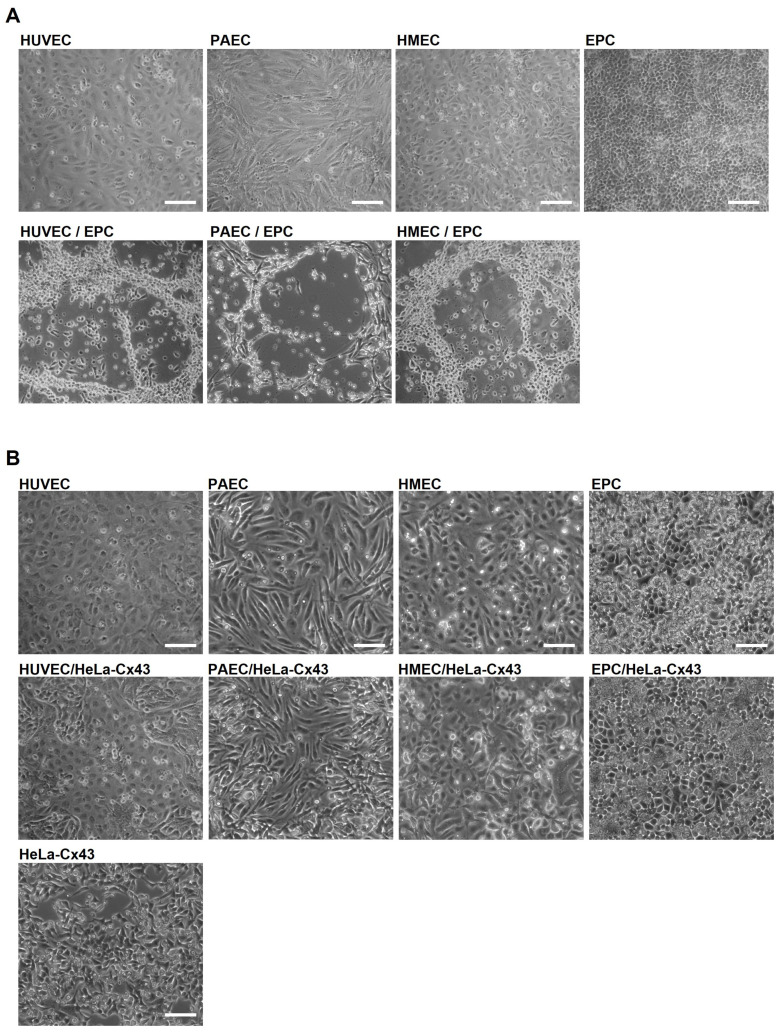
Co-cultivation of EC with EPC induces angiogenic network formation on uncoated cell dishes. (**A**) Representative images of HUVEC, PAEC and HMEC co-cultured with EPC or cultured as monolayers. EC/EPC co-cultivation induced an angiogenic network formation typically after 3–6 days (n = 7). Pictures were taken after 4 days. Scale bar: 100 µm. (**B**) Representative images of HUVEC, PAEC, HMEC and EPC cultivated as monolayers or co-cultured with HeLa-Cx43 and as control HeLa-Cx43 as monolayer. No angiogenic networks could be observed after 3–6 days in co-culture with HeLa-Cx43 cells. Representative pictures were taken after 4 days (n = 4). Scale bar: 100 µm.

To assess whether EPC can induce angiogenesis in co-cultures with EC in vitro, we cultivated embryonic EPC with different types of EC (human umbilical vein endothelial cells (HUVEC), porcine aortic endothelial cells (PAEC) or human microvascular endothelial cells (HMEC)) up to 6 days. Monocultures of EC or EPC alone on uncoated cell culture dishes showed a confluent cell layer, which was kept throughout the whole observation period. However, the co-cultivation of EC with EPC induced the formation of angiogenic networks on uncoated cell culture dishes (Figure 1A) after 3–6 days. These networks were formed in co-cultures of all three endothelial cell types with EPC (Figure 1A). We further investigated whether the spontaneous formation of angiogenic networks was specifically induced by co-cultivation of EC with EPC. Therefore, we co-cultivated EC with stably transfected HeLa cells, a non-endothelial cell line, overexpressing Cx43 (HeLa-Cx43). Indeed, the co-cultivation of EC with HeLa-Cx43 cells did not induce angiogenic network formation (Figure 1B), indicating the strong angiogenic potential of EPC and their capability to incorporate into the angiogenic networks.

### 2.2. Angiogenic Networks in Co-Cultures of EC and EPC Are Formed by Both Cell Types

**Figure 2:**
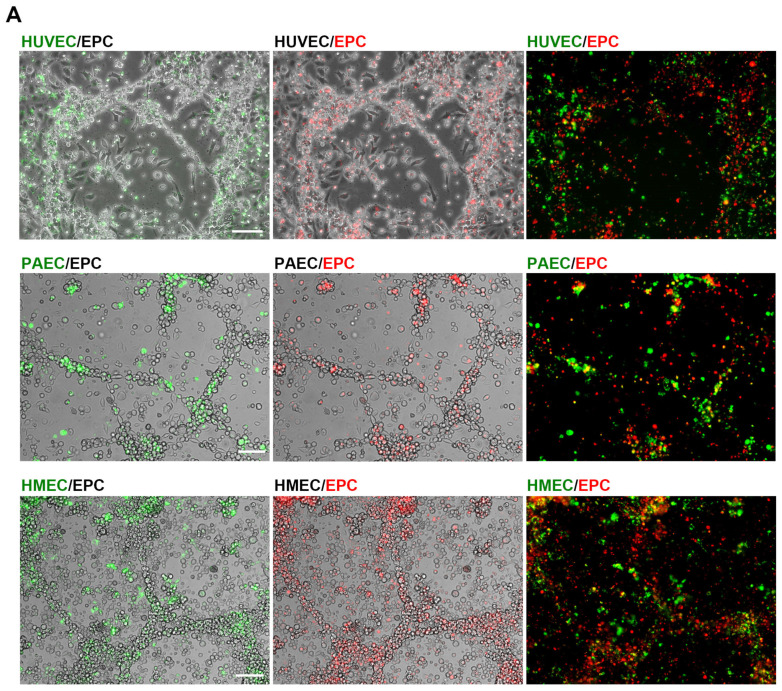

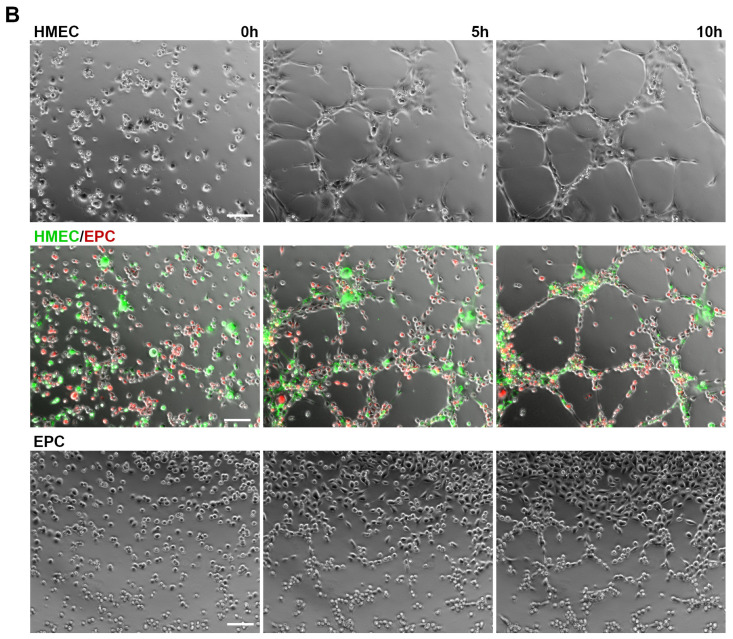
Angiogenic networks are formed by both cell types. (**A**) Angiogenic networks were formed on uncoated dishes in co-cultures of EC with EPC. Representative images of co-cultures of HUVEC, PAEC or HMEC, pre-labeled with the fluorescence dye PKH67 (green), and EPC fluorescently labeled with PKH26 (red) after 6 days (n = 4–6). Scale bar: 100 µm. (**B**) Angiogenic tube formation of EC, EPC and EC/EPC co-cultures on an angiogenic basement matrix (Geltrex). Representative images of HMEC, EPC or HMEC in co-culture with EPC at different time points (0 h, 5 h, 10 h) are shown (n = 6). Scale bar: 100 µm.

We further investigated whether EPC served as building blocks together with EC or merely as a source of angiogenic growth factors triggering EC to form angiogenic networks. Fluorescent labeling of EC (HUVEC, PAEC, HMEC) with the membrane labeling dye PKH67 (green) and EPC with PKH26 (red) prior to their co-cultivation revealed that apparently both cell types are contributing to the network formation recorded after 6 days (Figure 2A). To compare the angiogenic potential with an in vitro angiogenesis assay, we investigated the tube formation of EC and EPC as monocultures and in co-culture on an angiogenic basement matrix (Geltrex). In contrast to uncoated dishes, EC were able to form angiogenic networks on the basement matrix as monocultures (Figure 2B and Figure S1) as well as in co-culture with EPC.

### 2.3. EPC Communicate with EC via Functional Gap Junction Channels

**Figure 3:**
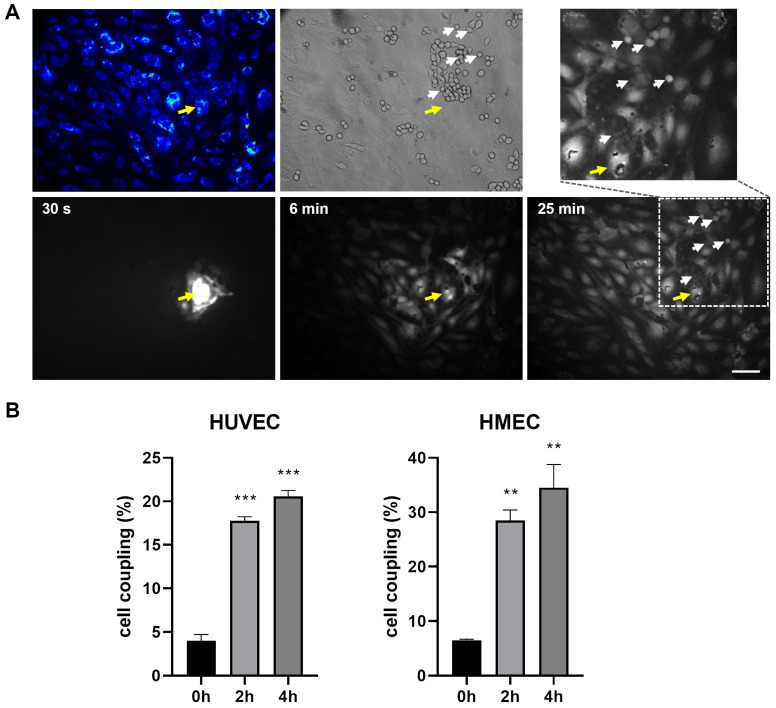

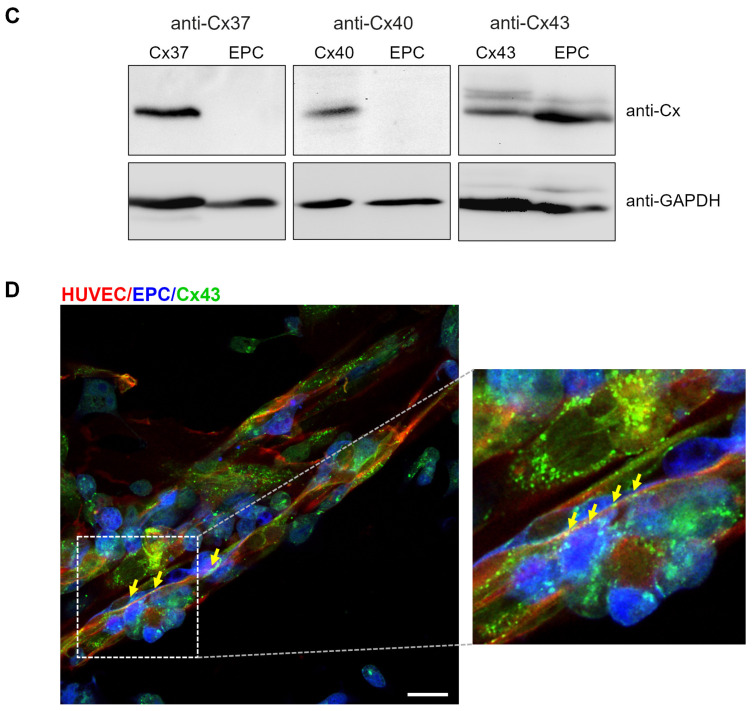
EC communicate with EPC via functional gap junction channels containing Cx43. (**A**) Representative images demonstrating the gap junctional dye transfer from HUVEC to EPC. HUVEC monolayers were stained with the fluorescent dye CMTMR (blue; top left) and then co-cultivated with EPC (n = 3 independent cell cultures). After 28 h of cocultivation, the fluorescent gap junction permeable dye Alexa Fluor 488 was injected into a single HUVEC cell (yellow arrow, injected cell not visible in the transmission channel). The diffusion of the fluorescent dye into surrounding HUVEC and EPC via gap junctions after 6 and 25 min is shown. A magnified image section marked by dotted lines depicts the fluorescent dye propagation into EPC (white arrows) after 25 min. The transmission picture (top middle) shows the distribution of unstained EPC (white arrows) on the HUVEC monolayer. Scale bar: 100 µm. (**B**) Gap junctional coupling of EPC with EC: HUVEC (left) and HMEC (right). HUVEC or HMEC were stained with the gap junction permeable dye calcein (0.04 µM, green fluorescence) and co-cultivated with PKH26-labeled EPC (red fluorescence) for 2 h and 4 h. Subsequently, the amount of green fluorescence in red-labelled EPC, indicating the gap junctional transfer of calcein, was quantified by FACS analysis. Data are represented as percentage of cell coupling, n = 2 (0 h), n = 4 (2 h, 4 h) independent cell cultures, (***) *p* < 0.001, (**) *p* < 0.01 versus each time point. (**C**) Western blot analysis of the expression of Cx37, Cx40 and Cx43 in EPC. HeLa cells expressing Cx37, Cx40 or Cx43 were used as positive control. Equal loading was confirmed by detection of GAPDH. (**D**) Immunofluorescence stainings of Cx43 in HUVEC/EPC co-cultures after 6 days. To distinguish EPC from HUVEC, GFP-transfected EPC (blue) were used. The co-cultures were stained for the endothelial specific cell marker CD31 (red) to specifically label HUVEC. Cx43 (green) was stained with a polyclonal antibody against Cx43. The right image shows a magnified section of the left image (dotted line) showing the membrane localization of Cx43 between both cell types (yellow arrows). Scale bar: 20 µm.

The close contact of EPC and EC in co-cultures allows both paracrine signaling and signaling via direct intercellular communication by gap junctions. To verify the gap junctional coupling, dye injection studies with Alexa Fluor 488 were performed after co-cultivation of HUVEC and EPC for 28 h. To distinguish both cell types, HUVEC were fluorescently labeled with CMTMR (Figure 3A, top right, blue) prior to their co-cultivation with unlabeled EPC. The gap junctional dye transmission from the injected single HUVEC (yellow arrow) to surrounding HUVEC and further to more distant EPC (white arrows) demonstrated the direct intercellular coupling of HUVEC with EPC via gap junctions (Figure 3A, bottom).

The gap junctional coupling between EC and EPC could further be confirmed and quantified by FACS analysis measuring de-novo gap junctions. For this, EPC were fluorescently labeled with PKH26 and co-cultured with EC pre-stained with the fluorescent dye calcein, which is transferred between cells via gap junctions. The gap junctional cell coupling, represented as percentage of double stained EPC, increased time dependently, indicating that EPC are functionally coupled to EC via gap junctions (Figure 3B). We therefore next analyzed the Cx expression in EPC. Western blot analyses revealed that of the vascular connexins Cx37, Cx40 and Cx43, which are expressed in mature EC [22], EPC specifically only express Cx43 and not Cx37 or Cx40 (Figure 3B), indicating that EPC couple with EC via Cx43 gap junctions. Immunofluorescence staining and subsequent confocal microscopy of HUVEC and EPC co-cultures after 6 days further demonstrated that Cx43 (green) was integrated into the membrane of neighboring HUVEC and EPC and thus enabling the coupling of both cells (Figure 3C).

### 2.4. Angiogenic Network Formation Is Not Induced by Conditioned Medium

**Figure 4:**
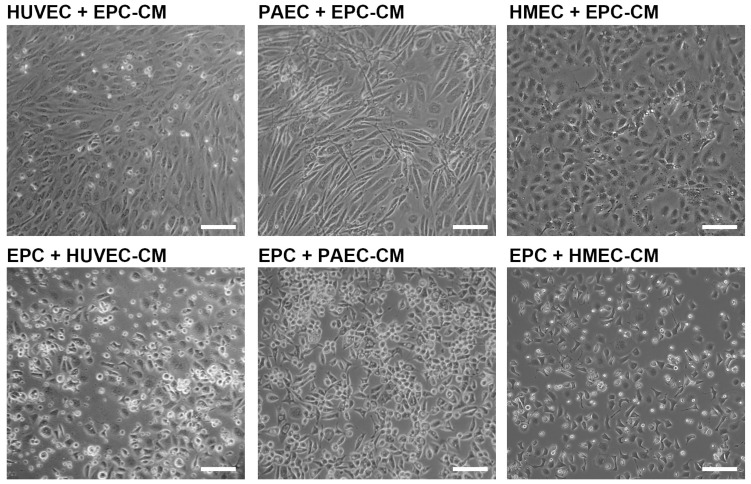
The angiogenic network formation is not induced by the incubation of EC with conditioned media of EPC. Supernatants of EPC (EPC-CM) grown in endothelial cell culture medium for 48 h were used to cultivate HUVEC, PAEC or HMEC for 5 days. Vice versa, EPC were cultured in conditioned media of HUVEC (HUVEC-CM), PAEC (PAEC-CM) or HMEC (HMEC-CM) for 5 days. The conditioned medium was changed every 2 days. Representative images demonstrate that the angiogenic network formation is not induced by angiogenic growth factors in the conditioned medium secreted by EPC (n = 4 independent cell cultures). Scale bar: 100 µm.

To investigate whether paracrine signaling (molecules secreted from EPC or EC) is sufficient to induce the angiogenic network formation, we next incubated EC with conditioned medium of EPC (EPC-CM) and EPC with conditioned medium of EC, respectively. Angiogenic networks in HUVEC, PAEC or HMEC (Figure 4) were not formed in the presence of EPC-CM. Likewise, conditioned medium of EC (HUVEC-CM, PAEC-CM, HMEC-CM) had no angiogenic effect on EPC (Figure 4). Only the co-cultivation of EC with EPC and the close contact of both cell types induced angiogenic network formation (Figure 1), indicating that the interaction of both is necessary for this.

### 2.5. Angiogenic Network Formation Is Impaired by Inhibitors of Gap Junctions

**Figure 5:**
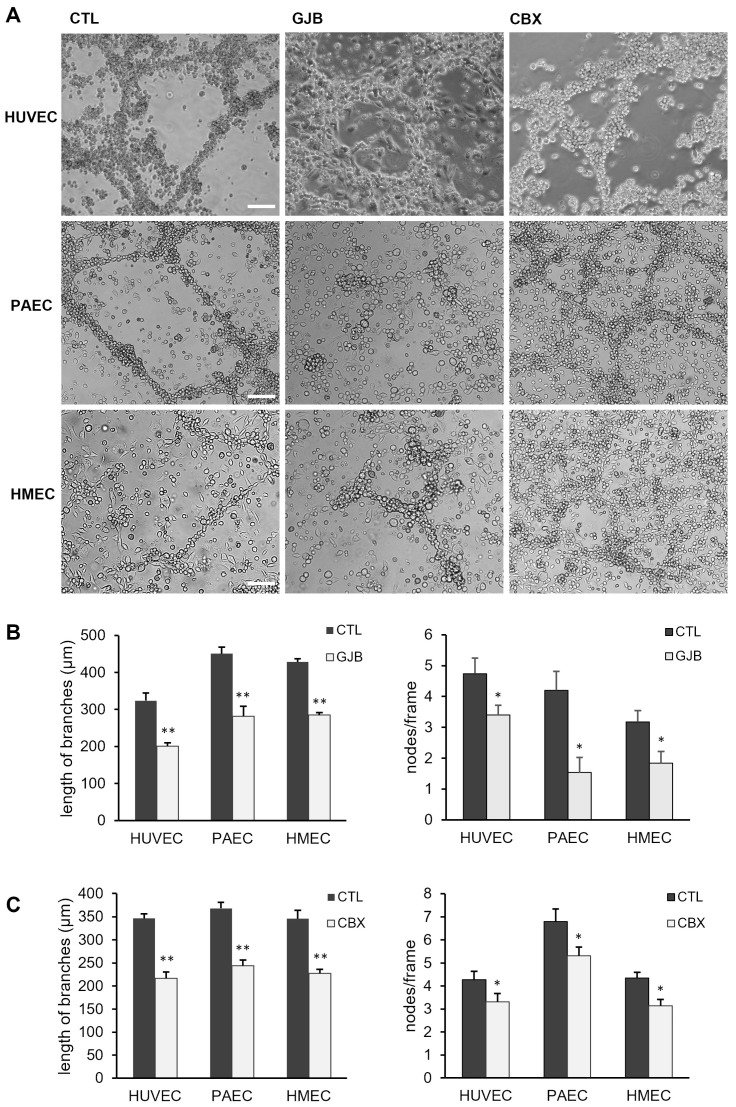
Angiogenic network formation is reduced by the inhibition of gap junctions. (**A**) Representative images of co-cultures of HUVEC, PAEC or HMEC with EPC which were treated with different gap junction blockers (GJB, CBX) or with the solvents alone as mock control (CTL) for 6 days. Blockers (GJB: 1 mM heptanol and 2.5 µM meclofenamic acid; CBX: 10 µM carbenoxolone) were added every second day. Scale bar: 100 µm. (**B**,**C**) The pharmacological inhibition of gap junctions by the treatment with GJB (**B**) or CBX (**C**) significantly reduced the length of branches and the number of branching points (nodes/frame) in angiogenic networks of HUVEC/EPC, PAEC/EPC and HMEC/EPC co-cultures (HUVEC/EPC: GJB: n = 4; CBX: n = 4; PAEC/EPC: GJB: n = 6; CBX: n = 9; HMEC/EPC: GJB: n = 6; CBX: n = 7; * *p* < 0.05, ** *p* < 0.01; GJB or CBX vs. CTL with the same number of n, respectively).

To identify whether the gap junctional coupling between EPC and EC affects the formation of angiogenic networks, long-term co-culture experiments were performed in the presence of different pharmacological gap junction blockers. To minimize the amount of the gap junction blockers and potential side effects, a combination of meclofenamic acid (2.5 µM) and heptanol (1 mM), indicated as GJB, was used. Both blockers are efficiently blocking Cx43 containing gap junctions [23]. The incubation of co-cultures of EC (HUVEC, PAEC, HMEC) and EPC with GJB impaired the formation of angiogenic networks (Figure 5A,B). In the quantitative analysis, the length of branches and the branching point intensity (nodes/per frame) were evaluated in the co-cultures. The length of branches (mean ± SEM; HUVEC: CTL: 323 ± 21 µm; GJB: 201 ± 9 µm; PAEC: CTL: 451 ± 18 µm; GJB: 281 ± 28 µm; HMEC: CTL: 428 ± 9 µm; GJB: 285 ± 7 µm) as well as the number of nodes/frame (mean ± SEM; HUVEC: CTL: 4.7 ± 0.5; GJB: 3.4 ± 0.3; PAEC: CTL: 4.2 ± 0.6; GJB: 1.5 ± 0.5; HMEC: CTL: 3.2 ± 0.4; GJB: 1.8 ± 0.4) within the angiogenic networks were significantly reduced in the presence of GJB compared to control co-cultures (CTL), which were only treated with the solvents (Figure 5B). To confirm these results, the co-cultures were alternatively treated with another non-selective pharmacological gap junction blocker, carbenoxolone (10 µM, CBX) [23]. The inhibitory effect of both pharmacological gap junction blockers (GJB, CBX) was first confirmed by scrape loading dye transfer assays (Figure S3). Similarly, inhibition of the gap junctional communication with CBX significantly reduced the length of branches (mean ± SEM; HUVEC: CTL: 346 ± 10 µm; CBX: 217 ± 14 µm; PAEC: CTL: 368 ± 13 µm; CBX: 244 ± 12 µm; HMEC: CTL: 346 ± 18 µm; CBX: 228 ± 9 µm) and the number of nodes (mean ± SEM; HUVEC: CTL: 4.3 ± 0.4; CBX: 3.3 ± 0.4; PAEC: CTL: 6.8 ± 0.5; CBX: 5.3 ± 0.4; HMEC: CTL: 4.3 ± 0.2; CBX: 3.1 ± 0.3) but somewhat less than GJB (Figure 5C). Additionally, to rule out potential unspecific toxic effects of the gap junction blockers GJB or CBX, we assessed the cell viability of monocultures and co-cultures after 3 and 6 days. The cell viability did not differ between the treated cultures and the appropriate mock controls (Figure S2). These results indicate that the gap junctional communication between EC and EPC is required for the formation of angiogenic networks in co-cultures of both cells and for the incorporation of EPC in these angiogenic network structures.

## 3. Discussion

The contribution of EPC in neovascularization during vascular remodeling processes has been investigated extensively [24,25,26]. However, whether the direct communication of EPC with EC via gap junctions contributes to the formation of angiogenic networks or whether a paracrine activation of EC by secreted proangiogenic factors of EPC alone is sufficient has not yet been studied in detail. The present study demonstrates that the gap junctional communication plays a crucial role for the spontaneous formation of angiogenic networks in co-cultures of EC and EPC and additionally the incorporation of EPC in newly formed vessels during angiogenesis. Noteworthy, co-cultivation of mature EC with embryonic EPC induced a spontaneous angiogenic network formation without other stimulating angiogenic factors and without three-dimensional gels of extracellular matrix (ECM) components like collagen gels, Geltrex or Matrigel. For the investigation of molecular mechanisms that are associated with angiogenesis, different three-dimensional (3D in vitro models are commonly used. These in vitro angiogenesis models aim to examine the ability of EC to migrate and differentiate into capillary-like structures by cultivating EC in 3D-gels of extracellular matrix proteins like collagen, fibrin, Geltrex or Matrigel (two extracellular matrix-based hydrogels with laminin as major component) [27,28] or in co-culture models of EC with supporting cells, e.g., fibroblasts, mesenchymal stem cells, or tumor cells [27,29,30,31]. The difference of these co-culture models to the EC/EPC co-cultures of this study is that EPC not only secrete factors like the above-mentioned supporting cells to stimulate the formation of angiogenic networks by EC but are also incorporated into the networks and are therefore an important part of these capillary-like structures. The ability of embryonic EPC to integrate into new blood vessels in vivo has already been demonstrated [3,21]. The spontaneous angiogenic network formation by co-cultivation of EC and EPC after 3–6 days in this study suggests an ECM deposition and assembly by EPC in these co-cultures as EC appear to express ECM proteins only intracellularly [32].

The embryonic EPC used in this study reportedly express and secrete factors that regulate angiogenesis [18,19]. Therefore, we further investigated whether this might be additionally important for the induction of the angiogenic network formation of EC. Our results clearly demonstrated that the incubation of EC with conditioned medium of EPC did not induce the formation of angiogenic networks in EC, suggesting that direct contact and interaction between EC and EPC is necessary for the angiogenic network formation.

Hence, we examined the Cx expression of EPC and whether they can couple with EC via gap junctions. The present results demonstrate that EPC selectively express Cx43 and are able to form functional gap junctions with EC. Most interestingly, the close contact of EC with EPC enabling Cx43 gap junctional coupling seems to be important for the formation of angiogenic networks in vitro, stimulating this process. Indeed, the pharmacological inhibition of the gap junctional communication significantly reduced the angiogenic network formation in EC/EPC co-cultures, but it was not prevented. The concentrations used of the gap junction blockers were kept as low as possible to avoid side effects but still high enough to block gap junction channels. However, the effect of the gap junction blockers (GJB or carbenoxolone) varied among the different EC/EPC co-cultures. Due to the different mechanisms of action, the impact of GJB (a combination of heptanol and meclofenamic acid) and CBX depends on the Cx expression and the membrane characteristics of the different EC. Heptanol influences the opening probability of gap junctions indirectly by affecting the structure of the lipid membrane, whereas CBX acts directly on the Cx protein. In addition, the blockers exhibit a different selectivity towards the Cx proteins. This could explain why their effects vary across the different EC types [23,33]. Nevertheless, these results clearly indicate that intercellular communication of EC and EPC via Cx43 is important for the angiogenic network formation in EC/EPC co-cultures but does not exclude additional so far unknown channel-independent contributing factors.

Several studies have repeatedly shown that Cx43 promotes angiogenesis e.g., in diabetic retinopathy [34], in pulmonary microvascular endothelial cells [35], or during reparative angiogenesis under chronic cerebral hypoperfusion [36], whereas Cx43 silencing inhibits this [14,15,16,36,37,38]. Most of these studies proposed that Cx43 exerts its proangiogenic effects—via its intracellular carboxyl tail through interaction with signaling proteins and their subsequent activation [15,35,39]. In our previous study, we demonstrated that knock-down of Cx43 significantly reduced endothelial cell migration of HMEC and impaired aortic vessel sprouting ex vivo [13]. In earlier studies, we demonstrated that Cx43 modulated cell motility and migration via its carboxyl tail and in a channel-independent manner [12,40,41]. Interestingly, Cx43 promotes angiogenesis also in EPC as the upregulation of Cx43 by inhibition of miR-206 promoted EPC proliferation and migration as well as angiogenesis [42], whereas the angiogenic potential was attenuated by Cx43 downregulation [16]. Whether this pro-angiogenic effect in EPC is channel dependent or channel independent is still unclear.

However, here we observed that gap junctional communication involving Cx43 between EPC and EC was necessary for the formation of angiogenic networks. Furthermore, the incorporation of both cell types was required for the spontaneous formation of these networks. The current findings are supported by previous work from others. For example, during blood vessel assembly, the gap junctional communication of mesenchymal progenitors with endothelial cells seems to be necessary for their subsequent differentiation into mural cells [43]. Similarly, the coupling of osteoprogenitor cells with endothelial cells via gap junctions induced their differentiation, and this was dependent on the expression of Cx43 [44]. Moreover, the heterogeneous interaction between cancer and endothelial cells via gap junctions has been shown to contribute to tumor progression and the formation of new blood vessels [45]. Another study showed that co-cultures of EPC and mesenchymal stem cells (MSCs) drove the differentiation of MSCs into SMCs via direct cell-to-cell contact and extracellular signal-regulated kinase (ERK) signaling [46].

Collectively, our results demonstrate that the direct interaction of EC with EPC via gap junction channels consisting of Cx43 is important for the incorporation of EPC and the spontaneous formation of angiogenic networks in EC/EPC co-cultures. Additionally, this study indicates that Cx43 promotes angiogenic network formation via gap junctional interaction of EC with EPC and thus in a channel-dependent way while at the same time modulating cell migration during angiogenesis in a channel-independent manner. Which molecules are transferred through gap junctions and what role they play in the control of angiogenesis is not yet fully understood. Recent research has highlighted the importance of Ca^2^⁺ signaling in the endothelium, particularly in the regulation of angiogenesis. Ca^2^⁺ waves are propagated across gap junctions between neighboring cells in a cell cluster [47]. It has been demonstrated that Ca^2^⁺ waves are important in the regulation of cell migration, which is one of the major processes during angiogenesis, and abolition of Ca^2^⁺ waves reduced migration as shown in smooth muscle cells [48]. In another study, Cx43-formed hemichannels were identified as important mediators of Ca^2^⁺ signaling during endothelial cell migration, providing an additional level of regulation beyond intercellular communication mediated by gap junctions [49]. In addition, gap junctions also participate in the transfer of small RNA molecules, such as miRNAs, between cells. These small RNA molecules have been shown to be important regulators of various cellular processes, including angiogenesis [50,51]. EPC are recruited from the bone marrow to the site of vascular injury, where they may incorporate and induce vascularization [1,2]. This has been demonstrated in different conditions, such as after myocardial infarction [52], in ischemic limbs [53] and in tumor angiogenesis [54]. Furthermore, conditions with reduced angiogenic activity, such as wound healing in diabetic patients, have been associated with a reduced number of EPC in these patients [55], further underlining their importance for (re)vascularization. Indeed, several studies have shown the promising potential of enhancing or promoting vascularization and vascular remodeling resulting in improved tissue perfusion in ischemic heart disease, peripheral arterial disease and diabetes by EPC-based therapies [52,55,56]. Thus, the present findings showing that EPCs are capable of forming angiogenic networks via Cx43-mediated gap junctions with endothelial cells may have significant implications for the further development of cell-based therapeutic strategies targeting vascular regeneration. The targeted delivery of EPCs to ischemic regions could enhance neovascularization, with Cx43-mediated gap junctions supporting the integration and functional organization of new capillary networks. Similar to CAR-T cell therapies, one may hypothesize that patient-derived EPC may be genetically manipulated to increase Cx43 expression. Through subsequent autologous EPC transplantation, the vascularization of ischemic tissues may then be enhanced by increasing the interaction and gap junctional communication between EPC and resident EC. Similarly, such an approach may be used to increase the perfusion of tumors, thus increasing the delivery and therefore success of anti-cancer drugs [57]. However, studies further investigating the exact mechanisms and factors involved in the gap junctional coupling between EPC and EC as well as the impact of this cell-cell communication is needed before the actual clinical potential of these findings can be properly estimated. Nevertheless, we deliver first evidence of gap junctional communication between EPC and EC being a critical mechanism in angiogenic network formation, thus contributing to the further unravelling of the underlying mechanisms and involved factors needed for EPC-induced angiogenic networks. This knowledge may contribute to the development of more specific and potent cell-based therapies in the future.

## 4. Materials and Methods

### 4.1. Cells and Culture Conditions

Human umbilical vein endothelial cells (HUVEC) were isolated as previously described [58], human microvascular endothelial cells (HMEC), provided by Ades et al. [59], and porcine aortic endothelial cells (PAEC), isolated as described by Gloe et al. [60], were cultured in Endothelial Cell Growth Medium MV (C-22020, Promocell, Heidelberg, Germany) containing 5% fetal calf serum (FCS) and supplemented with 1% penicillin/streptomycin (Thermo Fisher Scientific, Dreieich, Germany). Primary endothelial cells (HUVEC, PAEC) were used at passage 3–8 for the experiments. Mouse embryonic EPC, a kind gift from Dr. Antonis Hatzopoulos (Division of Cardiovascular medicine, Vanderbilt University Medical Center, USA), were cultured in Dulbecco’s modified Eagle medium (DMEM, Thermo Fisher Scientific) including 25 mM Hepes and supplemented with 20% fetal bovine serum (FBS, Avantor VWR, Darmstadt, Germany), 1 mM MEM non-essential amino acids (Thermo Fisher Scientific), 1% penicillin/streptomycin (Thermo Fisher Scientific, Dreieich, Germany), 2 mM L-glutamine (Thermo Fisher Scientific), 0.1 mM β-mercaptoethanol (AppliChem, Darmstadt, Germany). HeLa cells stably expressing Cx37, Cx40 or Cx43 were kindly provided by Dr. Klaus Willecke (University of Bonn, Germany) and cultivated in DMEM with 10% newborn calf serum (NBCS, Sigma Aldrich, Taufkirchen, Germany) and 1% penicillin/streptomycin (Thermo Fisher Scientific) supplemented with 1 µg/mL puromycin (Sigma Aldrich). All cells were maintained at 37 °C and 5% CO_2_.

### 4.2. Long-Term Co-Culture Experiments and Cell Viability Assay

Endothelial cells (HUVEC, HMEC, PAEC) were co-cultivated with EPC or as control with HeLa-Cx43 cells in a ratio of 3:1 in endothelial cell growth medium (Promocell) in uncoated 6-well-plates up to 6 days. Therefore, EC and EPC were detached from the culture plate with trypsin, resuspended in culture medium, and the cell number was determined. EC were then mixed with EPC in a ratio of 3:1 and co-cultured in endothelial medium. In parallel, EC and EPC were also cultivated as monocultures under the same conditions as a control. Images of co-cultures were taken with an inverted microscope (Zeiss Axio Observer with an AxioCam camera (Zeiss, Oberkochen, Germany) or Leica DM with a Flexacam camera (Leica Microsystems, Wetzlar, Germany)) at indicated time points to examine the angiogenic network formation. To investigate the angiogenic potential of secreted factors in cell culture supernatants, EC (HUVEC, PAEC, HMEC) were cultivated with supernatants of EPC, cultured for 48 h in endothelial cell growth medium (EPC-CM) and vice versa, EPC were incubated with conditioned medium of EC (HUVEC-CM; PAEC-CM; HMEC-CM) for the same time periods as co-cultures.

To inhibit the gap junctional communication, co-cultures of HUVEC/EPC, PAEC/EPC and HMEC/EPC were incubated with a combination of the pharmacological gap junction blockers meclofenamic acid (2.5 µM, dissolved in H_2_O; Sigma Aldrich) and heptanol (1 mM, dissolved in 100% ethanol; Sigma Aldrich), referred to as GJB, or with 10 µM carbenoxolone (CBX, dissolved in PBS, Sigma Aldrich). The gap junction inhibitors were added every second day. As control, co-cultures were treated with the same amounts of the respective solvents (CTL).

The cell viability was investigated in cell cultures of long-term co-culture experiments at different time points as indicated. The cell viability was assessed with the cell drop fluorescence cell counter (DeNovix, Wilmington, DE, USA) using acridine orange/propidium iodide staining (AO/PI) according to the manufacturer’s instructions (DeNovix). The fluorescent dye AO selectively stains nucleated living cells, whereas PI is impermeable for live cells and stains dead and dying cells. Briefly, the cells were trypsinized and resuspended in cell growth medium. The premixed AO/PI solution (DeNovix) was equilibrated to room temperature and briefly vortexed. The cell suspension (10 µL) was diluted 1:1 with the AO/PI solution (10 µL). The cell suspension-AO/PI-dilution was analyzed with the cell drop fluorescence cell counter, and the number of viable cells and dead cells determined.

### 4.3. Tube Formation Assay

To assess the angiogenic potential of EC and EPC, a tube formation assay was performed using Geltrex (Reduced-Growth Factor Basement-Membrane Matrix, Thermo Fisher Scientific). Prechilled 8-well μ-Slides (Ibidi, Gräfelfing, Germany) were coated with 200 µL of Geltrex per well and incubated at 37 °C for 1 h to allow polymerization. EC were stained with PKH 67 and EPC with PKH 26 as described below. The next day, the cells were trypsinized and resuspended in endothelial cell growth medium and counted with the cell drop fluorescence cell counter (DeNovix). EC and EPC were seeded as monocultures or co-cultures on the solidified Geltrex in a ratio of 3:1. The tube formation was observed with an inverted microscope (Zeiss Axio Observer) for 10 h by recording fluorescence and phase images every 10 min. Quantitative analysis of tube length was performed using ZenBlue (version 3.5, Zeiss).

### 4.4. Quantitative Evaluation of Angiogenic Networks

Co-cultures of EC and EPC were imaged with an AxioCam camera under an inverted microscope (Zeiss Axio Observer) to evaluate the angiogenic networks. The length of branches in µm was measured with the AxioVision software (version 4.8, Zeiss), and the branching points intensity (nodes per frame) were quantified (10 images/experiment evaluated). For each experiment, EC and EPC were seeded as monocultures in 6-well plates (two wells each), and EC were seeded with EPC as co-culture (two wells). To quantify the angiogenic network formation, at least 5–10 images/well were evaluated. For PAEC/EPC co-cultures: n = 6 independent cell cultures for CTL and GJB and n = 9 for CTL and CBX were evaluated. For HMEC/EPC co-cultures: n = 6 independent cell cultures for CTL and GJB and n = 7 for CTL and CBX were evaluated. For HUVEC/EPC co-cultures: n = 4 independent cell cultures for CTL and GJB as well as for CTL and CBX were evaluated.

### 4.5. Fluorescence Labelling of Live Cells with PKH Linkers

PKH fluorescent dyes provide the labeling of living cells over an extended period and were used to distinguish both cell types in long-term co-cultures. EC were stained with the fluorescent cell linker PKH67 (green fluorochrome, Sigma Aldrich) and EPC with PKH26 (red fluorochrome, Sigma Aldrich) prior to their co-cultivation. The cells were washed with PBS and incubated with the respective diluted PKH dye according to the manufacturer’s instructions for 5 min. After washing twice with culture medium, the cells were incubated with fresh culture medium for 24 h and were washed again prior to their co-cultivation.

### 4.6. Gap Junctional Dye Transfer in Co-Cultures

HUVEC monolayers were stained with 1 µM of the long-term fluorescent dye CMTMR (Thermo Fisher Scientific) according to the manufacturer’s instructions for 45 min, subsequently washed with phosphate-buffered saline (PBS) and incubated with fresh endothelial cell growth medium. The stained cells were washed again twice with growth medium at the next day and were then co-cultivated with unstained EPC for 28 h in endothelial cell growth medium. For coupling experiments, the gap junction permeable fluorescent dye Alexa Fluor 488 (3.5 mM, dissolved in 150 mM KCl; Molecular Probes, Thermo Fisher Scientific) was injected into a single CMTMR-stained endothelial cell (HUVEC). For this, a borsilicate glas micropipette (tip diameter < 1 µm) was mounted on a micromanipulator and connected to an injection system (Femtojet, Eppendorf, Hamburg, Germany) enabling an injection of the dye into a single cell for 0.5 s (tip pressure of 80 mmHg). Alexa Fluor 488 was excited at 488 nm, and images were captured at 515 nm (long pass emission filter) with a digital camera (Imago, Till photonics, Gräfelfing, Germany) and stored on a computer. The camera was mounted on an inverted microscope (Axiovert S 100, Zeiss, Göttingen, Germany) displaying areas of 640 µm × 640 µm (final magnification of 200). To demonstrate that the dye was spreading to all neighboring cells over time, fluorescence images were stored directly, 6 min and 25 min after the injection.

### 4.7. Analysis of Cell Coupling Using Flow Cytometry (FACS)

EC were grown to confluency in 12-wells plates and pre-stained with the fluorescent dye calcein-AM (0.04 µM, Thermo Fisher Scientific), for 30 min at 37 °C and 5% CO_2_ followed by several washing steps with PBS. After diffusion into the cells, intracellular esterases cleave the acetoxymethyl (AM) ester group, and the fluorescent dye calcein cannot further permeate the membrane, only allowing its propagation via gap junctions. EPC (dye acceptor cells) were pre-stained with PKH-26 Red fluorescent cell linker (2 µM, Merck, Darmstadt, Germany) for 5 min according to the manufacturer’s instructions. After 1 h incubation in cell growth medium, an EPC cell suspension of 280,000 cells/mL was added to the calcein-labelled EC (donor cells) and co-cultivated for 2 h and 4 h. Subsequently, the cells were washed and detached with trypsin. The cells were resuspended in cell growth medium, centrifuged (1200 rpm, 4 min), and the pellet resuspended in PBS. The cells were then analyzed by flow cytometry (10,000 cells) for double staining, indicating the gap junctional transfer of calcein into PKH 26-labeled EPC. The fluorescence was measured (CytoFLEX S, Beckman Coulter Life Sciences, Krefeld, Germany) at excitation/emission wave lengths of 494/517 nm (calcein) and 551/567 (PKH 26). The percentage of double stained cells (red and green) was determined as percentage of cell coupling.

### 4.8. Scrape-Loading Dye Transfer

The blocking efficiency of pharmacological gap junction inhibitors was investigated using the dye transfer after scrape loading technique as described before [61] being slightly modified. Briefly, the cells were seeded in 6-well plates and grown to confluency. Gap junctions were blocked with a mixture of 1 mM heptanol and 2.5 µM meclofenamic acid (GJB) or with 10 µM carbenoxolone (CBX) for 1 h. As control, the cells were treated with the same amount of the respective solvent. After washing twice with PBS, the cells were scraped with a razor blade and immediately incubated with 0.125% Lucifer Yellow (LY, Sigma Aldrich) dissolved in PBS for 5 min, with or without the respective gap junction blockers (GJB or CBX). Subsequently, the cells were washed twice with PBS and then fixed with 3.7% formaldehyde (Neolab, Heidelberg, Germany) dissolved in PBS for 15 min. Fluorescence images with individual scratches were recorded with an AxioCam camera (Zeiss) on an inverted microscope (Zeiss). The gap junctional dye transfer was evaluated using the software ImageJ (version 1.53t) as described by Begandt et al. [61] with slight modifications. For every image, 10 regions of interest (ROI) of 400 × 200 pixel (length × width) corresponding to 360 × 180 µm from the scrape line into the area of dye diffusion were set or in the area without dye diffusion for background subtraction, and the fluorescence intensity was plotted. Plot profiles were analyzed, and the gap junctional dye diffusion was determined.

### 4.9. Western Blot Analysis

Western blot analysis was performed as previously described [13]. Briefly, the cell lysates were prepared in Laemmli buffer [62] and boiled for 5 min. Proteins were size-separated by SDS-PAGE (10% or 8–16% Tris–Glycine gels, Serva, Heidelberg, Germany) and transferred to a Hybond-P membrane (Cytavia Amersham, Freiburg, Germany). Unspecific antibody binding was blocked by incubating the membranes in 5% skimmed milk (AppliChem) in PBS-0.1% tween (Sigma Aldrich) for 1 h. Membranes were incubated with the primary antibodies diluted in 5% bovine serum albumin (BSA, AppliChem) in PBS-0.1% tween overnight at 4 °C. The following primary antibodies anti-Cx37 (1:1000, Biotrend, Köln, Germany), anti-Cx40 (1:1000, Biotrend), anti-Cx43 (1:1000, Sigma Aldrich, Taufkirchen, Germany), anti-GAPDH (1:10,000, Merck) were used. After washing, the membranes were incubated with horseradish peroxidase coupled secondary antibodies (1:2000–1:5000; Merck) diluted in 5% skimmed milk powder in PBS-0.1% tween. Subsequently, the membranes were washed three times for 10 min, and bound antibodies were detected with enhanced chemiluminescence (ECL, AppliChem).

### 4.10. Immunofluorescence Stainings

Immunofluorescence staining was performed to analyze Cx43 localization and expression in EC/EPC co-cultures. EPCs were transfected with the vector pcDNA4-GFP as previously described [12]. Cells were seeded on glass coverslips coated with Collagen G (Biochrom, Berlin, Germany), placed in 24-well plates for 6 days until networks were formed. Cells were washed with PBS and fixed with 3.7% formaldehyde (Neolab, Heidelberg, Germany) for 15 min at room temperature. Following fixation, cells were permeabilized with 0.1% Triton X-100 (Sigma Aldrich) in PBS for 2 min and blocked with 1% BSA (AppliChem) in PBS for 1 h to prevent nonspecific antibody binding. The cells were incubated overnight at 4 °C with primary antibodies diluted 1:50 in 1% BSA/PBS. Antibodies targeting CD31 (Dako, Hamburg, Germany) and Cx43 (Sigma Aldrich) were used. After washing with PBS, cells were incubated for 1 h at room temperature with appropriate fluorophore-conjugated secondary antibodies (Alexa Fluor 546 (Thermo Fisher Scientific), Alexa Fluor 633 (Thermo Fisher Scientific)) diluted 1:200 in 1% BSA/PBS. Coverslips were mounted onto glass slides using Fluoromount-G mounting medium (SouthernBiotech, Birmingham, AL, USA) and stored at 4 °C in the dark until imaging. Fluorescent images were acquired using a confocal microscope (Leica, Microsystems, Wetzlar, Germany).

### 4.11. Statistics

For statistical computing, the data were analyzed using Sigma Plot 13.0 and Graph Pad Prism (software version 9). The number of each experiment was at least n ≥ 3 independent cell cultures, and the exact number of experiments is stated in the respective figure legend. Unpaired *t*-tests were performed for comparison between two groups with normal distribution. For more than two comparisons, the one-way analysis of variance, ANOVA, followed by pairwise multiple comparisons was applied. For timeline experiments, two-way ANOVA (mixed method) was used. Results were depicted as mean ± standard error of mean (SEM), and differences were considered significant at *p*-values smaller than 0.05 (*p* < 0.05).

## Data Availability

The data sets generated during the current study are available from the corresponding author upon reasonable request.

## Supplementary Materials

The following supporting information can be downloaded at: https://www.mdpi.com/article/10.3390/ijms26104827/s1.

## Abbreviations

The following abbreviations are used in this manuscript:

GJB	gap junction blockers heptanol and meclofenamic acid
Cx	connexin/s
EC	endothelial cells
EPC	endothelial progenitor cells
CBX	carbenoxolone
