# Supplementary material for: Gap Junctional Interaction of Endothelial Progenitor Cells (EPC) with Endothelial Cells Induces Angiogenic Network Formation In Vitro
Source: Int J Mol Sci. 2025 May 18;26(10):4827. doi: 10.3390/ijms26104827 (PMC12112054; doi:10.3390/ijms26104827)
Supplement: Supplementary file 1 [file ijms-26-04827-s001.zip › ijms-3516338-supplementary.pdf]

Supplement

Gap junctional interaction of endothelial progenitor cells (EPC) with endothelial cells induces angiogenic network formation *in vitro*

Christina Buchberger [1], Petra Kameritsch [2], Hanna Mannell [1], Heike Beck [3], Ulrich Pohl [3] and Kristin Pogoda [1,*]

1 Physiology, Institute of Theoretical Medicine, Faculty of Medicine, University of Augsburg, 86159 Augsburg, Germany

2 Walter Brendel Centre of Experimental Medicine, University Hospital, Ludwig-Maximilians-University, 81377 Munich, Germany

3 Walter Brendel Centre of Experimental Medicine, Biomedical Center Munich, Ludwig-Maximilians-University, 82152 Planegg, Germany

* Correspondence: kristin.pogoda@med.uni-augsburg.de; Tel.: +49 821 598 71121

Figure S1:

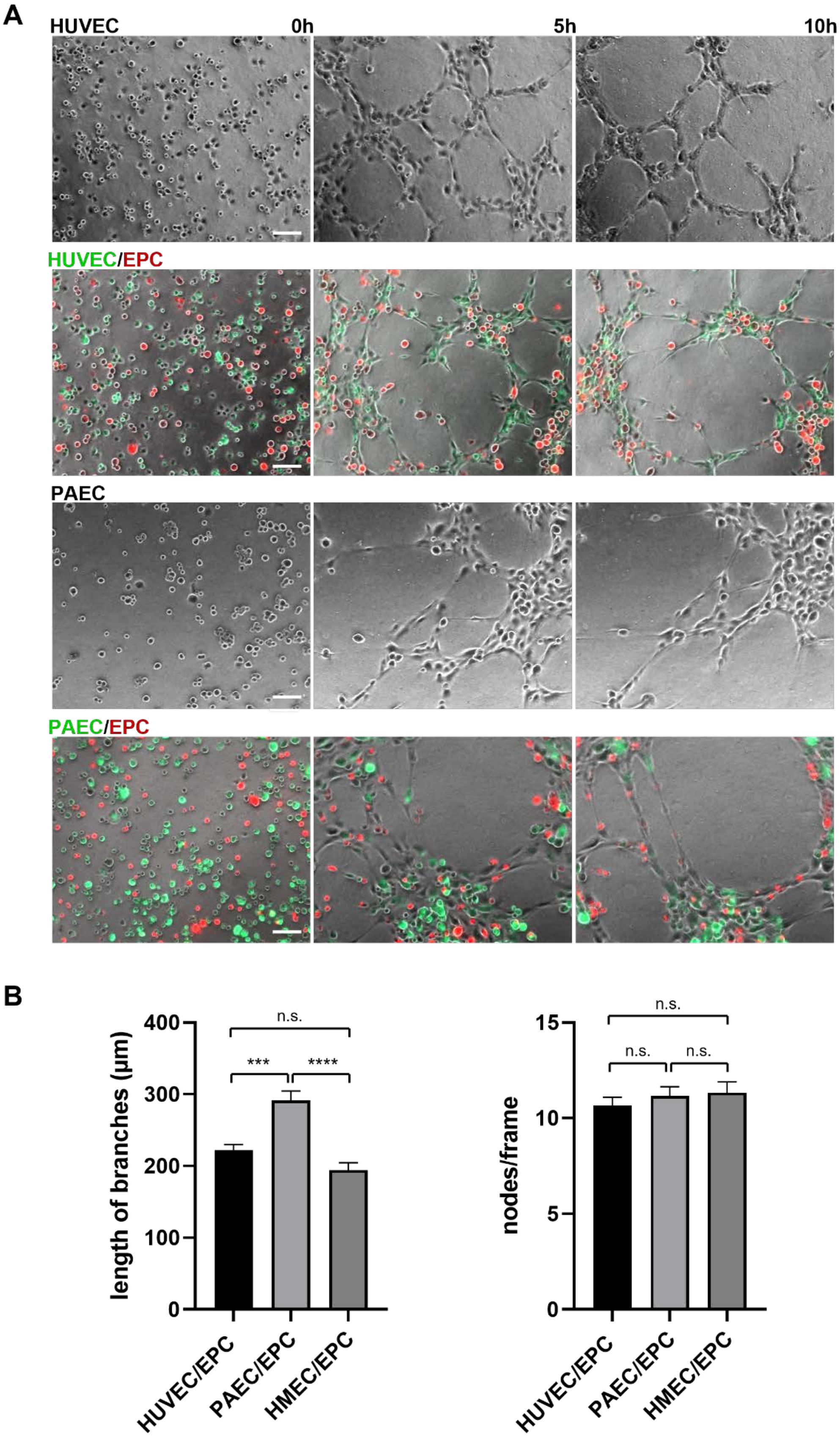

A
HUVEC
0h
5h
10h
HUVEC/EPC
PAEC
PAEC/EPC
B
length of branches (µm)
400
300
200
100
0
n.s.
***
****
HUVEC/EPC
PAEC/EPC
HMEC/EPC
nodes/frame
15
10
5
0
n.s.
n.s.
n.s.
HUVEC/EPC
PAEC/EPC
HMEC/EPC

Figure S2:

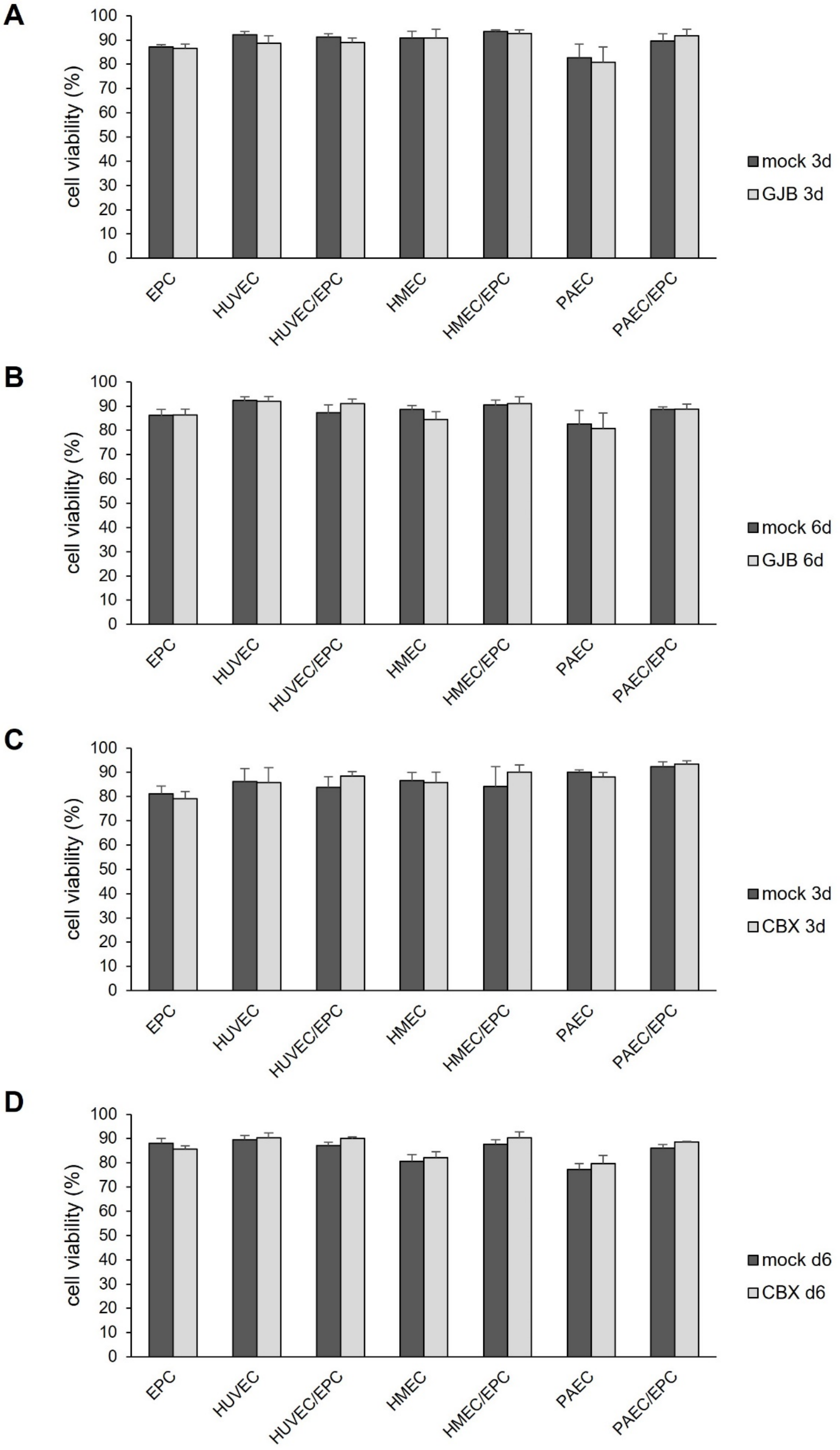
A
cell viability (%)
100
90
80
70
60
50
40
30
20
10
0
EPC
HUVEC
HUVEC/EPC
HMEC
HMEC/EPC
PAEC
PAEC/EPC
mock 3d
GJB 3d
B
cell viability (%)
mock 6d
GJB 6d
C
cell viability (%)
mock 3d
CBX 3d
D
cell viability (%)
mock d6
CBX d6

Figure S3:

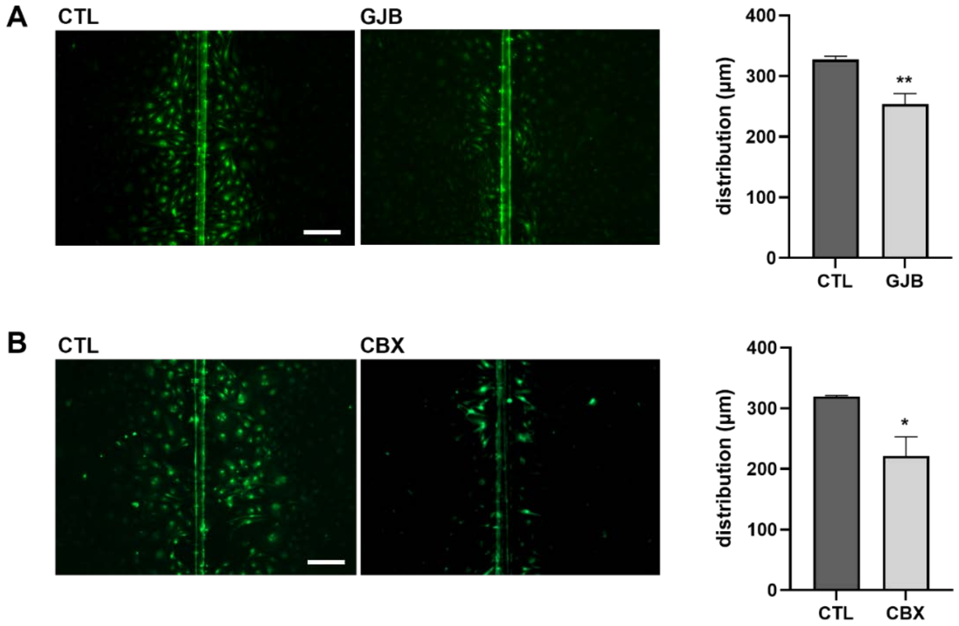
A
CTL
GJB
400
300
200
100
0
distribution (μm)
**
CTL
GJB
B
CTL
CBX
400
300
200
100
0
distribution (μm)
*
CTL
CBX

Figure legends:

Figure S1: Angiogenic tube formation of EC and EC/EPC co-cultures on Geltrex. (A) Representative images of HUVEC and PAEC as monoculture or in co-culture with EPC at different time points (0 h, 5 h, 10 h) are shown. Scale bar: 100 µm. (B) The length of branches (measured after 10 h) varied slightly among the different EC/EPC co-cultures with highest values for PAEC/EPC. The number of nodes per frame was very similar between the different EC/EPC co-cultures. The results are given as mean ± SEM (n=6 different experiments, ****$p<0.0001$, ***$p<0.001$, n.s.: not significant).

Figure S2: The pharmacologic inhibition of gap junctions did not affect the cell viability of long-term monocultures and co-cultures. Cell viability of EPC, HUVEC, HUVEC/EPC, HMEC, HMEC/EPC, PAEC and PAEC/EPC treated with GJB (1 mM heptanol and 2.5 µM meclofenamic acid) or with the solvents (100% ethanol, H_2O) alone as mock control after 3 days (A) and after 6 days (B) or treated with 10 µM carbenoxolone or the solvent alone (PBS) after 3 days (C) and after 6 days (D). The treatment with GJB (A, B) or CBX (C, D) did not affect the cell viability (GJB: n=3-7; CBX: n=3-6 independent cell cultures). The results are given as mean ± SEM.

Figure S3: Gap junction coupling is inhibited by GJB and CBX. EC monolayers (PAEC) were incubated with GJB (GJB: 1 mM heptanol and 2.5 µM meclofenamic acid), 10 µM CBX or with the solvents alone as mock control (CTL) for 1 hour. Gap junctional coupling was examined by the SLDT technique. Representative fluorescent images show the diffusion of LY in GJB-treated PAEC monolayers (A), CBX-treated PAEC (B) and in control-treated PAEC monolayers. Scale bar: 200 µm. The inhibition of gap junctions was evaluated quantitatively by measuring the distance of the dye diffusion. The treatment with GJB (A) or CBX (B) significantly decreased the gap junction coupling (GJB: n=5; ** $p<0.01$; CBX: n=4; * $p<0.05$).
